# Endothelial-Dependent and Independent Vascular Relaxation Effect of Tetrahydropalmatine on Rat Aorta

**DOI:** 10.3389/fphar.2019.00336

**Published:** 2019-04-16

**Authors:** Zhong-Yan Zhou, Wai-Rong Zhao, Wen-Ting Shi, Ying Xiao, Zi-Lin Ma, Jin-Gui Xue, Lun-Qing Zhang, Qing Ye, Xin-Lin Chen, Jing-Yi Tang

**Affiliations:** ^1^Longhua Hospital, Shanghai University of Traditional Chinese Medicine, Shanghai, China; ^2^State Key Laboratory of Quality Research in Chinese Medicine, Institute of Chinese Medical Sciences, University of Macau, Macau, China; ^3^Shuguang Hospital, Shanghai University of Traditional Chinese Medicine, Shanghai, China; ^4^Faculty of Health Sciences, University of Macau, Macau, China; ^5^Cardiac Rehabilitation Center of Longhua Hospital, Shanghai University of Traditional Chinese Medicine, Shanghai, China

**Keywords:** Tetrahydropalmatine, vasorelaxation, calcium influx, endothelium function, potassium channel, calcium channel, calcium sensitization

## Abstract

Tetrahydropalmatine (THP) is an active natural alkaloid isolated from *Corydalis yanhusuo* W.T. Wang which has been widely used for treating pain and cardiovascular disease in traditional Chinese medicine. Previous studies suggested THP have various pharmacological effects in neural and cardio tissue while the vascular reactivity of THP was not fully established. The present study found that THP relaxed rat aorta which contracted by phenylephrine (Phe), KCl, and U46619. The vascular relaxation effect of THP was partially attenuated by PI3K inhibitor wortmannin, Akt inhibitor IV, endothelial nitric oxide synthetase (eNOS) inhibitor L-NAME, guanylate cyclase inhibitors and the mechanical removal of endothelium. Also, the eNOS substrate L-arginine reversed the inhibition effect of L-NAME on THP-induced vascular relaxation. THP also induced intracellular NO production in human umbilical vein endothelial cells. However, Pre-incubation with β-adrenergic receptor blocker propranolol, angiotensin II receptor 1 (AT1) inhibitor losartan, angiotensin II receptor 2 (AT1) inhibitor PD123319 or angiotensin converting enzyme inhibitor enalapril enhanced the vascular relaxation effect of THP. THP did not affect the angiotensin II induced vascular contraction. Cyclooxygenase-2 (COX2) inhibitor indomethacin did not affect the vascular relaxation effect of THP. Furthermore, pre-treatment THP attenuated KCl and Phe induced rat aorta contraction in standard Krebs solution. In Ca^2+^ free Krebs solution, THP inhibited the Ca^2+^ induced vascular contraction under KCl or Phe stress and reduced KCl stressed Ca^2+^ influx in rat vascular smooth muscle cells. THP also inhibited intracellular Ca^2+^ release induced vascular contraction by blocking Ryr or IP3 receptors. In addition, the voltage-dependent K^+^ channel (Kv) blocker 4-aminopyridine, ATP-sensitive K^+^ channel (KATP) blocker glibenclamide and inward rectifying K^+^ channel blocker BaCl_2_ attenuated THP induced vascular relaxation regardless of the Ca^2+^-activated K^+^ channel (KCa) blocker tetraethylammonium. Thus, we could conclude that THP relaxed rat aorta in an endothelium-dependent and independent manner. The underlying mechanism of THP relaxing rat aorta involved PI3K/Akt/eNOS/NO/cGMP signaling path-way, Ca^2+^ channels and K^+^ channels rather than COX2, β-adrenergic receptor and renin-angiotensin system (RAS). These findings indicated that THP might be a potent treatment of diseases with vascular dysfunction like hypertension.

## Graphical Abstract

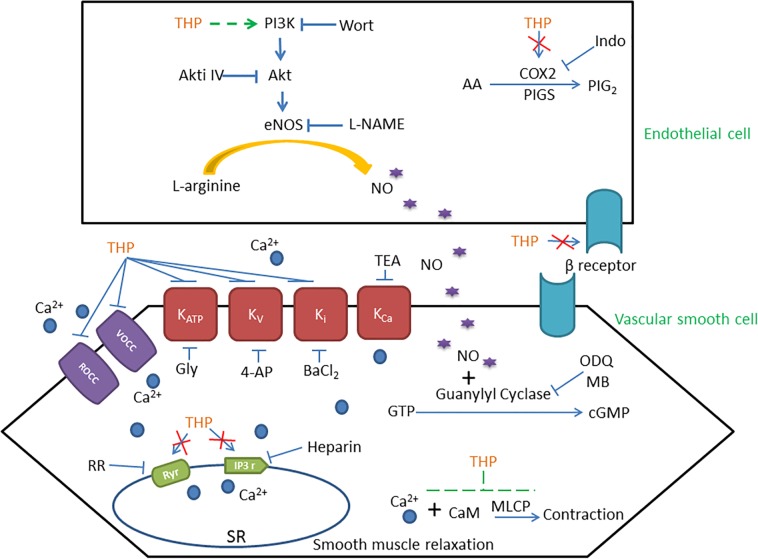

## Introduction

Hypertension is a worldwide disease, while the prevalence of hypertension arising and the control rate is less than 20% in China (Armario et al., 2017; Lu et al., 2017). Vascular function plays a vital role in the maintenance of normal blood pressure. Endothelial function, intracellular and extracellular ions in particular balance intracellular Ca^2+^ concentration and calcium sensitization primary control vascular function (Wang et al., 2015; Garcia et al., 2017; Zhou et al., 2017). Previous studies have suggested that Chinese medicine ameliorated endothelial dysfunction, vascular contractility, and vasodilation, which were important in hypertension therapy. Blood-Letting therapy, *Qigong Ba Duan Jin* and *Tai Chi* excises provided an alternative approach for hypertension therapy (Sun and Buys, 2015; Xiao et al., 2016; Ma et al., 2018; Xiong et al., 2019). Chinese herb medicine, such as *Ilex hainanensis* Merr., *Longdanxiegan* Decoction, also enhanced the effectiveness of anti-hypertension drug (Xiong et al., 2018; Yang et al., 2018). Thus, traditional Chinese medicine plays a vital role in the prevention and treatment of hypertension, especially in China.

**FIGURE 1:**
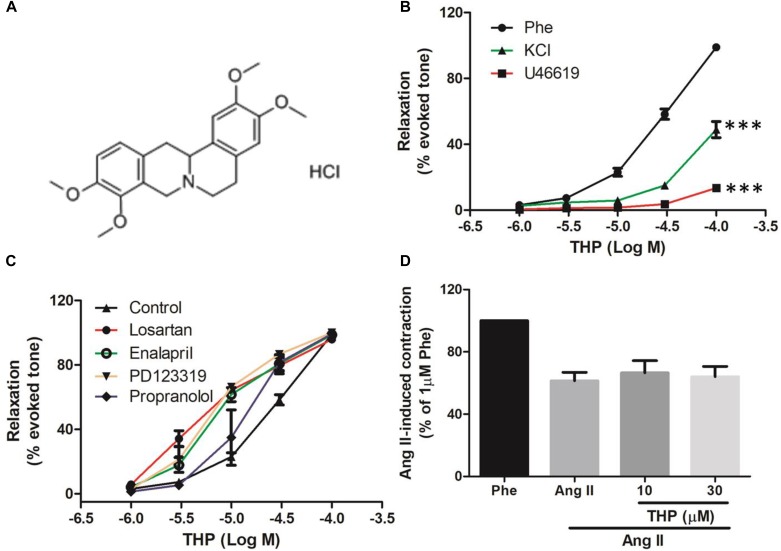
Tetrahydropalmatine (THP) induced vascular relaxation in rat aorta. **(A)** The chemical structure of Tetrahydropalmatine Hydrochloride (THP, PubChem CID: 6602555). **(B)** Rat aortic rings were pre-contracted by phenylephrine (Phe, 1 μM), U46619 (30 nM), or KCl (60 mM) and various concentration of THP (1, 3, 10, 30, and 100 μM) were cumulative applied to induce vascular relaxation. **(C)** Aortic rings were pre-incubated propranolol (1 μM), Losartan (1 μM), Enalapril (1 μM), or PD123319 (1 μM) for 30 min followed by the addition of Phe (1 μM) to induce contraction and relaxed by cumulative addition of THP (1, 3, 10, 30, and 100 μM). **(D)** Aorta rings were pre-incubated THP (10 and 30 μM) for 30 min then followed by the addition of Ang II (3 μM) to induce vascular contraction. Vascular relaxations or contractions were presented as percentage of the evoked tone. Results were means ± SEM. ^∗∗∗^*P* < 0.001 versus control group.

Tetrahydropalmatine (THP, Figure 1A) is a natural alkaloid isolated from *Corydalis yanhusuo* W.T. Wang which has been widely used in traditional Chinese medicine for treating various pains and cardiovascular disease (Han et al., 2012; Kang et al., 2016). Previous studies have indicated that THP presented multiple pharmacological effects on cardio and neural tissues, such as cardioprotection, neuroprotection, anti-oxidant, anti-apoptosis, and anti-inflammation (Wu et al., 2010; Zhang et al., 2015, 2018). THP improved memory impairment, protected the blood brain barrier and cerebral ischemia-reperfusion injuries in experimental mice models (Mao et al., 2015; Cao et al., 2018; Sun et al., 2018). Chueh et al. found THP could induce hypotension and bradycardia though inhibition of the 5-HT2 and/or D2-receptor in the hypothalamus in rats (Chueh et al., 1995; Fu et al., 1995). THP presented numerous benefits in blood vessels as well. THP reduced the inflammation process of monocyte binding to endothelium by down regulation of ICAM-1 and VCAM-1 in endothelial cells (Yang et al., 2015). THP also inhibited the progression of aortic aneurysms (AAs) though the suppression of matrix metalloproteinase and monocyte chemotactic protein-1 in rat (Wang et al., 2018). Moreover, THP relaxed rabbit atrial strips in a calcium channel blocker like manner (Sun and Li, 1989) and inhibited the kinetic activity of Kv1.5 channels expressed in HEK293 cells (Li et al., 2017). These results suggested that THP presented various pharmacological effects in the vascular system and is potent for anti-hypertension therapy. However, the vascular reactivity of THP and the action mechanism has not yet been fully understood.

In present study, we examined the vascular relaxation effects of THP under different contractors in rat aorta. The roles of endothelium, vascular smooth muscle cell and related signaling pathway and ion channels in the vascular relaxation effect of THP were also evaluated.

## Materials and Methods

### Chemicals

Acetylcholine (Ach), phenylephrine (Phe), Indomethacin (Indo), and angiotensin II (Ang II) were bought from Sigma-Aldrich (St. Louis, MO, United States). 9,11-dideoxy-9α,11α-methanoepoxy Prosta-glandin F2α (U46619), L-NAME, nifedipine, 1H-[1,2,4]-oxadiazolo-[4,3-alpha]-quinoxalin-1-one (ODQ) and Losartan, Enalapril, PD123319 were from Cayman Chemical (MI, United States). Fura-2 was supplied by Beyotime Biotechnology (Shanghai, China). THP hydrochloride (purity by HPLC ≥98.0%) was bought from Chengdu Must Bio-Technology Co., Ltd. (Chengdu, China). THP was dissolved in dimethyl sulfoxide (DMSO) while other drugs were prepared in distilled water.

### Animals

Male Wistar rats weighting 250 ± 20 g were supplied by the Laboratory Animal Service Center, Shanghai University of Traditional Chinese Medicine. All experiments described below were in accordance with the Animal Experimentation Ethics Committee, Shanghai University of Traditional Chinese Medicine.

### Artery Preparation

Rats were killed by carbon dioxide suffocation. After scarification, aortas were quickly isolated and immersed in oxygenated (95% O_2_/5% CO_2_) chilled Krebs solution with the following composition (mM):119 NaCl, 4.7 KCl, 2.5 CaCl_2_, 1 MgCl_2_, 25 NaHCO_3_, 1.2 KH_2_PO_2_, and 11 D-glucose. Fat and connective tissues were removed carefully. Then aortas were cut into ring segments with a length of 3–4 mm. The endothelium was mechanically removed by gently rubbing the internal surface of the ring using stainless steel wire.

### Measurement of Isometric Vascular Tone

Isometric tension of aortic rings was recorded in 15-ml organ bath (Techman Software Co., Ltd., Chengdu, China). The organ chambers were filled with Krebs solution bubbled with 95% O_2_ and 5% CO_2_ at 37°C. Each ring was stretched to 1.5 g resting tension. Before each experiment, the rings were equilibrated for 60–90 min and stimulated with 60 mM KCl at least three times to obtain a reproducible maximal contractile response. The integrity of endothelium was assessed by the ability of Ach (10 μM) to induce more than 80% relaxation of rings pre-contracted with Phe (1 μM). In endothelium-denuded rings, the relaxation to Ach was less than 10%. Ca^2+^-free Krebs solution was prepared by the omission of CaCl_2_ and the addition of 0.5 mM EGTA. In order to ensure the repeatability of the study, 11–13 g was selected as the inclusion criterion of pre-contractile force.

### Experimental Procedures

#### Effect of THP on Vascular Contraction Induced by Phe, KCl, and U46619

Phe (1 μM), KCl (60 mM), or U46619 (30 nM) was applied to contract rat aorta and the cumulative concentration response of THP (1, 3, 10, and 30, 100 μM) was examined. The experiments were repeated with the addition of the solvent DMSO at 1:1000 v/v (volume of DMSO per volume of final solution volume) to the contracted arteries, though this did not result in any relaxation (data not shown), thus verifying that the relaxations observed are most likely due to the action of THP. Relaxations were expressed as the percentage of the plateau contraction.

#### Role of β-Adrenergic Receptor and Renin-Angiotensin System (RAS) in THP-Induced Vascular Relaxation

Aortic rings were pre-incubated with β-adrenergic receptor blocker propranolol (1 μM), angiotensin II receptor 1 (AT1) inhibitor losartan (1 μM), angiotensin II receptor 2 (AT2) inhibitor PD123319 (1 μM), or angiotensin converting enzyme inhibitor enalapril (1 μM) for 30 min, then the concentration-response to accumulative addition of THP (1–100 μM) was studied in aortic rings pre-contracted by Phe (1 μM). Aortic rings were pre-incubated with THP (10 and 30 μM) for 30 min which was followed by the addition of Ang II (3 μM) to induce vascular contraction. Relaxations or contractions were expressed as the percentage of the Phe-induced contraction.

#### Role of Endothelium, PI3K/Akt/eNOS/NO/cGMP, and COX2/PGI2 Signaling Pathway in THP-Induced Vascular Relaxation

Aortic rings were incubated with guanylate cyclase inhibitor Methylene blue (MB, 5 μM), nitric oxide synthase (NOS) inhibitor L-NAME (100 μM), PI3K inhibitor Wortmannin (Wort, 0.5 μM), Akt inhibitor IV (0.5 μM), cyclooxygenase-2 (COX2) inhibitor Indomethacin (Indo, 1 μM), or NO-sensitive guanylyl cyclase inhibitor ODQ (3 μM) for 30 min. Alternatively, we performed mechanical removal of endothelium (-endo), and then the concentration-response to accumulative addition of THP (1–100 μM) was studied in aortic rings pre-contracted by Phe (1 μM). L-arginine (L-Arg, 100 μM) was used to argue the effect of L-NAME on THP induced aorta relaxation. Relaxations were expressed as the percentage of the plateau contraction.

#### Effect of THP on High K^+^ or Phe-Induced Vascular Contraction

Aortic rings were pre-treated with THP (10, 30, and 100 μM) or DMSO (1:1000 v/v) which served as solvent control for 30 min, then followed by KCl (60 mM) or Phe (1 μM)-induced vascular contraction. The plateaued contraction force values were recorded. The L-type Ca^2+^ channel blocker nifedipine (100 nM) was used as positive control. Vascular tensions were expressed as the percentage of the plateau contraction induced by KCl (60 mM) or Phe (1 μM) in the control group.

#### Effect of THP on Extracellular Ca^2+^-Induced Vascular Contraction

Aortic rings were challenged with high K^+^ (60 mM KCl) or Phe (1 μM) containing Ca^2+^-free Krebs solution. The cumulative concentration-response curves of CaCl_2_ (0.1–3 mM) were obtained after incubation with THP (10 and 100 μM), nifedipine (100 nM) or DMSO (1:1000 v/v) for 30 min. The contractile responses to CaCl_2_ were expressed as the percentage of the plateau contraction induced by KCl (60 mM) or Phe (1 μM) in standard Krebs solution.

#### Roles of K^+^ Channels in THP-Induced Vascular Relaxation

To elucidate the roles of K^+^ channels in THP-mediated relaxation, intact or endothelium denuded aortic rings were pre-incubated with the voltage-dependent K^+^ channel (Kv) blocker 4-aminopyridine (4-AP, 100 μM) and ATP-sensitive K^+^ channel (KATP) blocker glibenclamide (Gly, 1 μM), Ca^2+^-activated K^+^ channel (KCa) blocker tetraethylammonium (TEA, 1 mM), or inward rectifying K^+^ channel blocker (BaCl_2_, 100 μM) for 30 min, then followed by Phe (1 μM) to reach a plateaued contraction and cumulative concentration-response to THP (1–100 μM) was measured. Relaxations were expressed as the percentage of the plateau contraction.

#### Roles of Intracellular Ca^2+^ Storage in THP-Induced Vascular Relaxation

The rat aortas were immersed in Ca^2+^ free Krebs solution and pre-incubated with THP (10, 30, and 100 μM) for 30 min, then vascular contracted by KCl (60 mM) or Phe (1 μM). The Ca^2+^ was released from the intracellular Ca^2+^ store though Ryr and IP3 receptors in the endoplasmic reticulum. Thus, the endothelium denuded rat aortas were pre-treated with Ryr inhibitor ruthenium red (RR, 10 μM) or IP3 receptor heparin (5 mg/ml) for 30 min, then vascular contracted by Phe (1 μM) and relaxed by the accumulative addition of THP (1–100 μM). Relaxations were expressed as the percentage of the plateau contraction.

### Human Umbilical Vein Endothelial Cell (HUVEC) Culture and Detection of Intracellular Nitric Oxide (NO)

The HUVEC cell line was purchased from the American type culture collection (ATCC) and cultured in cultured in F-12K medium supplemented with 0.1 mg/ml heparin, 0.05 mg/ml endothelial cell growth supplement (ECGS), 10% (v/v) FBS and 1% penicillin/streptomycin at 37°C in a humidified atmosphere of 5% CO_2_ in air. HUVECs were stained with the NO probe DAF-FM DA (5 μM) for 30 min followed by NO imaging every 10 s with a real-time cell imaging system. After 5 min baseline intracellular NO recorded, HUVECs were treated with THP (25 and 100 μM) or 0.01% DMSO and continually record to 1 h. The data calculated as the ratio of fluorescent signal value at indicated time (F1) and the fluorescent signal value at the 0 time point (F0).

### Rat Vascular Smooth Muscle Cell A7r5 Culture and Detection of Intracellular Calcium

The A7r5 cell line was purchased from the ATCC and cultured in Dulbecco’s modified Eagle’s medium (DMEM, Gibco) supplemented with 10% FBS (Gibco). A7r5 cells were incubated at 37°C in a humidified atmosphere of 5% CO_2_ in air. A7r5 cells were stained by the calcium indicator Fura-2 (1 μM) for 15 min in Krebs solution and followed by the incubation of THP (100 μM) or nifedipine (100 nM) for 30 min in Ca^2+^ free Krebs solution containing KCl (60 mM). Then, the CaCl_2_ (2.5 mM) was added to trigger the Ca^2+^ influx. Nifedipine served as a positive control. The values of 550 nm emission were recorded at the 340 nm excitation and 380 nm excitation in the same time by calcium imaging system (Nikon Ti-E, Japan). The intracellular calcium amount was measured as ratio 340 nm/380 nm (R). The calcium imaging system recorded 60 s to obtain the stable baseline. The change of ratio 340 nm/380 nm value (ΔR) was calculated as ratio 340 nm/380 nm value after the addition of CaCl_2_ for 2 min (R_1_) minus ratio 340 nm/380 nm value before the addition of CaCl_2_ (R_0_).

### Data Analysis

Data was mean ± SEM from at least three vascular rings from different rats. The vascular relaxation degree was presented as a percentage of the evoked contraction. Data was analyzed using GraphPad Prism software (version 5.0). The half-maximum effective concentration (EC_50_) means the concentration of THP that induced 50% of maximal relaxation (E_max_). The negative logarithm of the EC_50_ (pD2) was calculated from the concentration-response curves by non-linear regression (curve fit). The Student’s unpaired *t*-test or analysis of variance (ANOVA) was used for statistical evaluation of difference between two groups. *P* < 0.05 indicated a significant difference.

## Results

### Tetrahydropalmatine (THP) Concentration Dependently Relaxed Rat Aorta Pre-contacted by Phenylephrine (Phe), KCl, or U46619

In order to examine the vascular relaxation effect of THP (Figure 1A) caused by different contractors, rat aorta was pre-contracted by KCl, Phe, or U46619 then relaxed by cumulative addition of THP. As the results shown in Figure 1B and Table 1, THP relaxed rat aortas in a concentration-dependent manner while the relaxation response cures were not consistent between vascular challenged by different contractors. The vascular relaxation effect of THP was most effective in the aorta rings pre-contracted by Phe and the corresponding maximal relaxation values (E_max_) and negative logarithm of the half-maximum effective concentration (pD2) were 98.97 ± 1.31 and 4.48 ± 0.07%, respectively (Table 1). In addition, pre-incubation with β-adrenergic receptor blocker propranolol, angiotensin II receptor 1 (AT1) inhibitor losartan, angiotensin II receptor 2 (AT2) inhibitor PD123319 or angiotensin converting enzyme inhibitor enalapril enhanced the vascular relaxation effect of THP in Phe-induced vascular contraction (Figure 1C and Table 1). Pre-treatment THP not affected the vascular contraction induced by angiotensin II (Figure 1D). So, the vascular relaxation effect of THP was not like relating to β-adrenergic receptor and renin-angiotensin system (RAS).

**Table 1:** Characteristics of THP (1–100 μM)-induced vascular relaxation under different conditions in intact (endo) and endothelium denudated (-endo) rat aorta.

Treatment	pD2	E_max_ (%)
**endo**		
Control (Phe)	4.48 ± 0.07	98.97 ± 1.31
60 mM KCl	5.50 ± 4.27	48.99 ± 4.89^∗∗∗^
U46619	2.77 ± 49.53	13.46 ± 1.35^∗∗∗^
Propranolol	4.84 ± 0.10^∗∗∗^	99.36 ± 0.44
Losartan	6.13 ± 0.45^∗∗∗^	95.89 ± 1.98
Enalapril	5.14 ± 0.08^∗∗∗^	98.67 ± 1.43
PD123319	5.21 ± 0.07^∗∗∗^	100.02 ± 0.51
L-NAME	3.76 ± 1.35	81.48 ± 3.11^∗∗∗^
Indo	1.40 ± 28.52	95.33 ± 1.47
Indo + L-NAME	4.21 ± 0.26	91.54 ± 1.95
L-NAME+Arg	4.70 ± 0.32	99.40 ± 0.56
L-NAME+ODQ	3.54 ± 12.2	68.53 ± 6.77^∗∗∗^
MB	4.33 ± 0.26	58.74 ± 3.31^∗∗∗^
ODQ	4.49 ± 0.10	60.46 ± 7.25^∗∗∗^
Wort	4.62 ± 0.04	94.11 ± 2.13^∗∗^
Akti IV	4.75 ± 0.06^∗∗^	98.77 ± 0.50
BaCl_2_	5.20 ± 0.18^∗^	97.75 ± 1.09
Gly	4.56 ± 0.35	89.34 ± 6.60^∗∗^
TEA	4.88 ± 0.23	99.95 ± 1.62
4-AP	4.59 ± 0.45	84.94 ± 1.26^∗^
**-endo**		
Control (-endo)	3.14 ± 3.44	85.58 ± 3.05^∗∗^
BaCl_2_	4.36 ± 3.05	82.33 ± 4.93
Gly	4.15 ± 1.65	77.48 ± 8.73
TEA	4.09 ± 1.81	81.66 ± 4.59
4-AP	3.81 ± 3.46	83.60 ± 7.18


*Results were means ± SEM. ^∗^*P* < 0.05, ^∗∗^*P* < 0.01, and ^∗∗∗^*P* < 0.001 versus control (Phe) group.*

### THP-Induced Vascular Relaxation Was Dependent on Endothelium and Related to PI3K/Akt/eNOS/NO/cGMP Signaling Pathway, but Not Involved in COX2/PGI_2_

**FIGURE 2:**
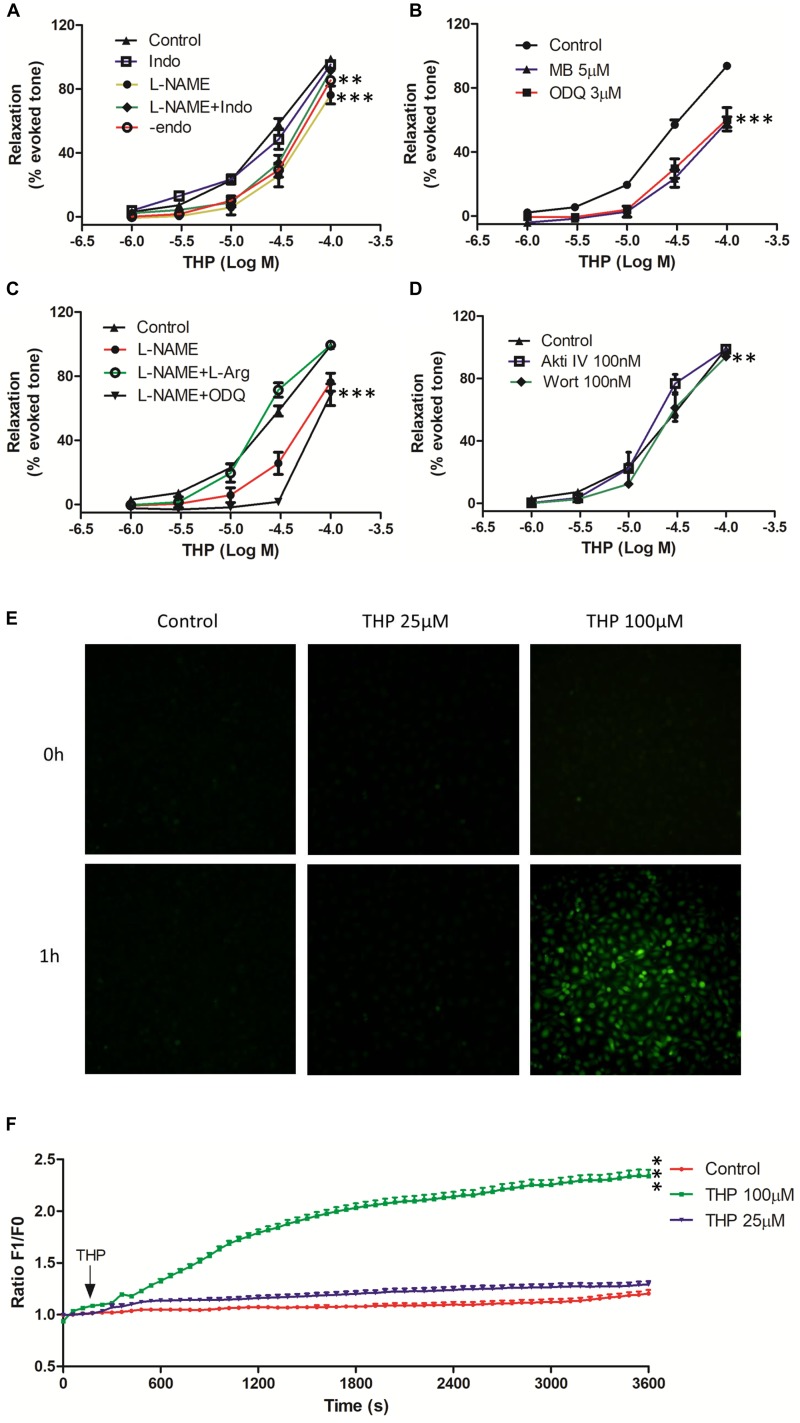
Tetrahydropalmatine-induced vascular relaxation was dependent on endothelium and related to nitric oxide (NO) production and bioavailability. The rat aortas were contracted by Phe (1 μM). **(A)** The effects of eNOS inhibitor (L-NAME, 100 μM), COX2 inhibitor (Indo, 1 μM) or mechanical removal of endothelium (-endo) on THP-induced vascular relaxation. **(B)** The effects of soluble guanylate cyclase inhibitors ODQ (3 μM) and methyl blue (MB, 5 μM) on THP-induced vascular relaxation. **(C)** The addition of L-arginine (L-Arg, 100 μM) reversed the inhibition effect of L-NAME on THP-induced vascular relaxation in Phe pre-contracted rat aorta. L-NAME (100 μM) together with ODQ (3 μM) reduced THP-induced vascular relaxation. **(D)** The effects of PI3K inhibitor wortmannin (Wort, 0.1 μM) and Akt inhibitor IV (Akti IV, 0.1 μM) on THP-induced vascular relaxation. **(E)** Representative images of NO staining of HUVECs under different conditions. HUVECs were stained with NO indicator DAF-FM DA (5 μM) for 30 min followed by NO real-time record using a real-time cell imaging system. After 5 min baseline NO recorded, HUVECs were treated with THP (25 and 100 μM) or DMSO (1:1000 v/v) and continually record to 1 h. **(F)** Statistic graph presented the real-time NO fluorescence change in HUVECs. F1 and F0 represented the fluorescent signal value at the indicated time point and 0 time point, respectively. Vascular relaxation presented as percentage of the evoked tone. Results were means ± SEM of more than three experiments. ^∗∗^*P* < 0.01, ^∗∗∗^*P* < 0.001 versus control group.

Nitric oxide (NO) and Prostacyclin (PGI_2_) were main endothelium dependent relaxing factors (EDRF). In present study, we found pre-incubation with endothelial nitric oxide synthase (eNOS) inhibitor L-NAME, soluble guanylyl cyclase inhibitor ODQ, guanylyl cyclase inhibitor methylene blue (MB), and mechanical removal of endothelium (-endo) suppressed the vascular relaxation effect of THP (Figure 2A,B). The addition of L-arginine (L-Arg) reversed the inhibition effect of L-NAME in THP induced vasodilation. Also, the combination of L-NAME and ODQ significantly inhibited THP-induced vascular relaxation (Figure 2C). Regarding the E_max_, there was no significant difference between the L-NAME-pre-treated group and the mechanical removal of endothelium (-endo) group (Table 1). Moreover, the PI3K inhibitor wortmannin (Wort) and Akt inhibitor IV (Akti IV) slightly suppressed THP-induced vascular relaxation (Figure 2D and Table 1). Also, THP induced intracellular NO production in HUVECs (Figure 2E,F). These results indicated that the vascular relaxation effect of THP was dependent on endothelium and mediated by NO synthesis and bioavailability via PI3K/Akt/eNOS/NO/cGMP signaling pathway. However, cyclooxygenase-2 (COX2) inhibitor indomethacin (Indo) did not affect the vascular relaxation effect of THP. The vascular relaxant inhibition level by L-NAME pre-treatment or mechanical removal of endothelium (-endo) was coincident with the combination treatment of Indo and L-NAME (Figure 2A and Table 1). So, the secretion of PGI_2_ by endothelium was not likely to be involved in the vascular relaxation effect of THP. The vascular relaxation effect of THP was dependent on endothelium. The underlying mechanism of THP relaxing rat aorta was related to PI3K/Akt/eNOS/NO/cGMP signaling pathway and not dependent on the COX2/PGI_2_ signaling.

### THP Reduced Vascular Tension and Intracellular Ca^2+^ Concentration by Blocking Calcium Channels

**FIGURE 3:**
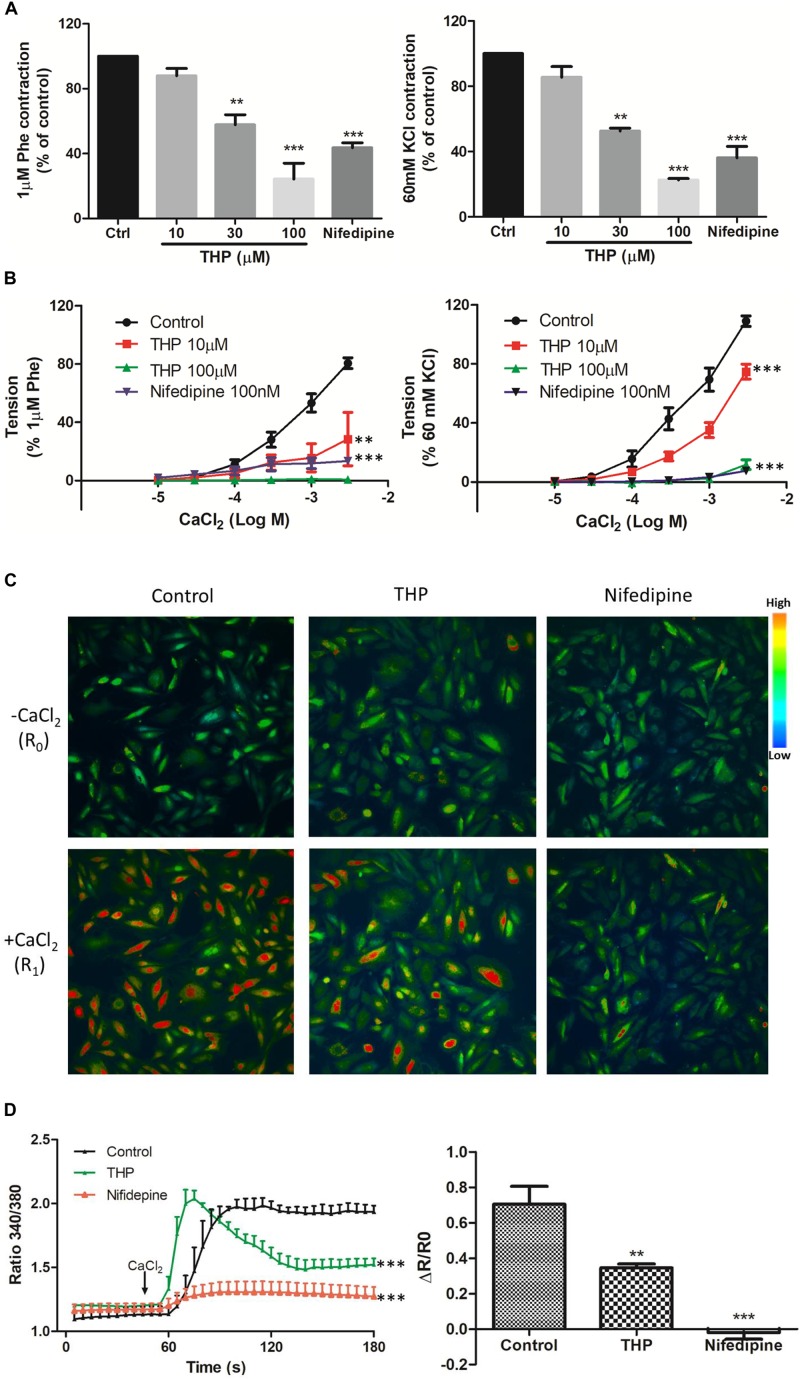
Tetrahydropalmatine-induced vascular relaxation was dependent on inhibition of Ca^2+^ influx. **(A)** Pre-treatment THP (10, 30, and 100 μM) inhibited KCl (60 mM) or Phe (1 μM) induced contraction of aorta in a concentration dependent manner. **(B)** Concentration-response curves showed pre-treatment THP (10 and 100 μM) inhibited CaCl_2_ (0.01–3 mM) induced contraction of aorta in Ca^2+^-free Krebs solution containing KCl (60 mM) or Phe (1 μM). **(C)** Representative images of calcium imaging. In A7r5 cells, pre-treatment THP (100 μM) or nifedipine (100 nM) inhibited the Ca^2+^ influx triggered by the addition of CaCl_2_ (2.5 mM) in Ca^2+^ free Krebs solution containing KCl (60 mM). **(D)** Time-course response and summarized graph of ratio 340 nm/380 nm (R). The change of ratio 340 nm/380 nm value (ΔR) was calculated as ratio 340 nm/380 nm value after the addition of CaCl_2_ for 2 min (R_1_) minus ratio 340 nm/380 nm value before the addition of CaCl_2_ (R_0_). Nifedipine served as positive control. Vascular contraction presented as percentage of the evoked tone. Results were means ± SEM of more than three experiments. ^∗∗^*P* < 0.01, ^∗∗∗^*P* < 0.001 versus control group.

Phe and KCl induced vascular contraction by inducing Ca^2+^ influx though voltage-dependent calcium channels (VDCC) and receptor-operated calcium channels (ROCC) (Niazmand et al., 2014; Zhou et al., 2017). We found that aortas pre-incubated THP significantly inhibited Phe or KCl-induced contraction in a concentration-dependent manner (Figure 3A). In line with these results, pre-incubation THP also inhibited the cumulative addition of CaCl_2_ triggered the vascular tension of aorta in Ca^2+^-free depolarizing Krebs solution containing KCl or Phe (Figure 3B). In rat vascular smooth muscle cell A7r5, we also observed that THP reduced Ca^2+^ influx which was trigged by CaCl_2_ in Ca^2+^-free Krebs solution containing 60 mM KCl (Figure 3C,D). L-type calcium channel blocker nifedipine serve as positive control in these experiments. These results revealed that THP suppressed vascular tension and reduced the intracellular Ca^2+^ concentration by blocking calcium channels.

### THP Suppressed Intracellular Ca^2+^ Release-Induced Vascular Tension by Blocking Ryr and IP3 Receptors

**FIGURE 4:**
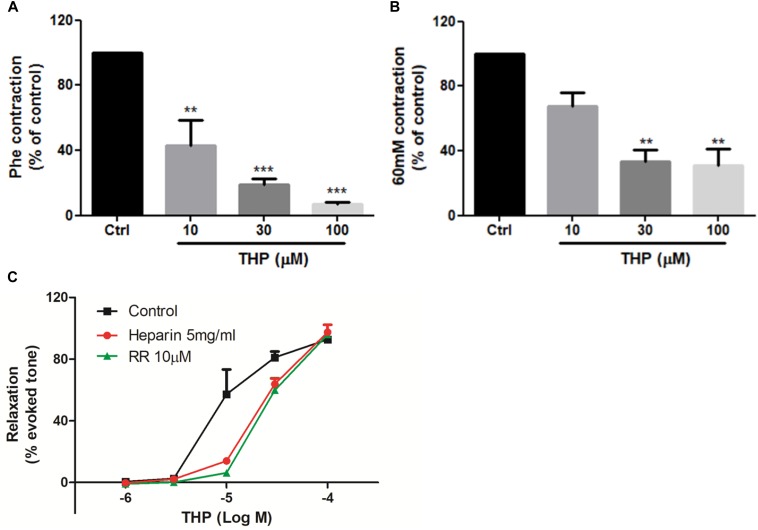
Tetrahydropalmatine inhibited intracellular Ca^2+^ release-induced vascular contraction. **(A)** Pre-treatment THP (10, 30, and 100 μM) suppressed KCl (60 mM) induced contraction of aorta in Ca^2+^-free Krebs solution. **(B)** Pre-treatment THP (10, 30, and 100 μM) suppressed Phe (1 μM) induced vascular contraction of aorta in Ca^2+^-free Krebs solution. **(C)** Pre-incubation Ryr inhibitor ruthenium red (RR, 10 μM) or IP3 receptor inhibitor heparin (5 mg/ml) attenuated THP induced rat aorta relaxation in Phe (1 μM) induced vascular tension in Ca^2+^-free Krebs solution. Vascular contraction and relaxation presented as percentage of control or evoked tone. Results were means ± SEM of more than three experiments. ^∗∗^*P* < 0.01, ^∗∗∗^*P* < 0.001 versus control group.

The data in Figure 3 indicated THP relaxed rat aorta by blocking Ca^2+^ channels and inhibited Ca^2+^ influx. Whether THP affected cellular internal Ca^2+^ release is unknown. KCl and Phe-induced cellular depolarizing lead to Ca^2+^ transient releases from intracellular calcium stores in endoplasmic reticulum (Ratz et al., 2005; Qin et al., 2014). So, we immersed aortic rings in Ca^2+^ free Krebs solution to avoid Ca^2+^ influx from extracellular medium. We found pre-incubation THP suppressed KCl or Phe-induced contraction of aorta in Ca^2+^-free Krebs solution (Figure 4A,B). Intracellular Ca^2+^ mainly released from Ca^2+^ store through IP3-dependent channels and the Ca^2+^ signaling amplified by ryanodine receptors (Ryr) (del Valle-Rodríguez et al., 2003). Consistently, pre-treatment Ryr inhibitor ruthenium red (RR) or IP3 receptor inhibitor heparin reduced the vasorelaxation effect of THP in Ca^2+^ free Krebs solution (Figure 4C and Table 2). Thus, the underlying mechanism of THP inhibiting the vascular tension induced by intercellular Ca^2+^ release to cytosol was blocking Ryr and IP3 receptors.

**Table 2:** The effects of IP3 and Ryr receptor inhibitor on THP (1–100 μM)-induced vascular relaxation in Ca^2+^ free Krebs solution.

Treatment	pD2	E_max_ (%)
Control	5.09 ± 0.08	92.744 ± 1.06
Ruthenium red (RR)	4.59 ± 0.03^∗^	97.50 ± 4.77
Heparin	4.63 ± 0.03^∗^	95.96 ± 2.23


*Results were means ± SEM. ^∗^*P* < 0.05 versus control group.*

### THP Relaxed Rat Aorta by Partially Inhibition of K^+^ Channels

**FIGURE 5:**
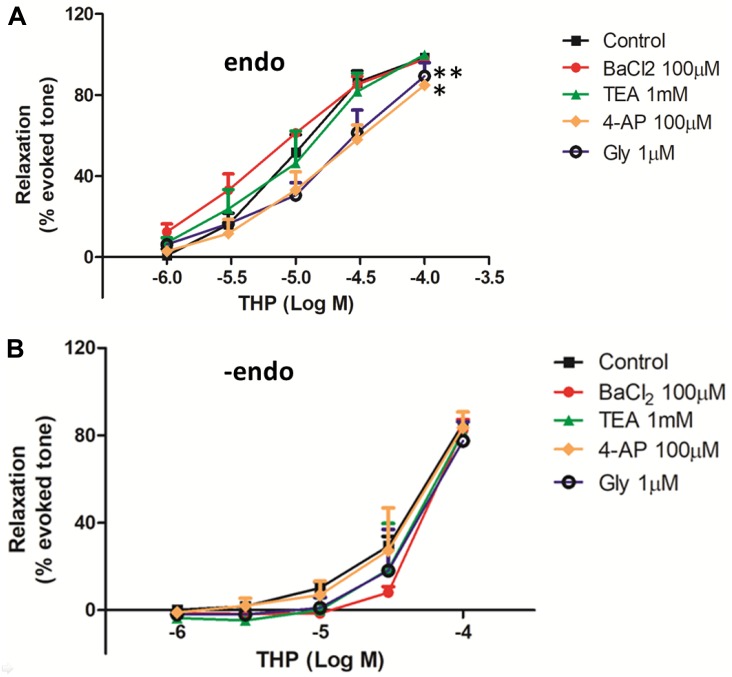
The effect of K^+^ channels on THP-induced vascular relaxation. The rat aortas were with intact endothelium (endo) **(A)** or mechanical remove the endothelium (-endo) **(B)**. The aortic rings were pre-incubated with the voltage-dependent K^+^ channel (Kv) blocker 4-aminopyridine (4-AP), ATP-sensitive K^+^ channel (KATP) blocker glibenclamide (Gly), Ca^2+^-activated K^+^ channel (KCa) blocker tetraethylammonium (TEA), and inward rectifying K^+^ channel blocker (BaCl_2_) on the indicated concentration for 30 min, then the vascular contracted with Phe (1 μM) and relaxed by the accumulative addition of THP (1–100 μM). Vascular relaxation presented as a percentage of the evoked tone. Results were means ± SEM of more than three experiments. ^∗^*P* < 0.05, ^∗∗^*P* < 0.01 versus control group.

In this study, we evaluated the effects of K^+^ channel blockers on the vascular relaxationinduced by THP in both intact and endothelium denuded rat aortas. The results from Figure 5A and Table 1 show that the voltage-dependent K^+^ channel (Kv) blocker 4-aminopyridine (4-AP), ATP-sensitive K^+^ channel (KATP) blocker glibenclamide (Gly), and inward rectifying K^+^ channel blocker (BaCl_2_) attenuated THP induced vascular relaxation in intact rat aorta regardless of Ca^2+^-activated K^+^ channel (KCa) blocker tetraethylammonium (TEA). However, K^+^ channel blockers did not affect the vascular relaxation effect of THP in endothelium denuded rat aorta (Figure 5B and Table 1). So, we could conclude that the underlying mechanism of vasodilatory effect of THP was partially though blocking K^+^ channels.

## Discussion

The present study examined the vascular reactivity of Tetrahydropalmatine (THP, Figure 1A) in rat aorta. We demonstrated that THP relaxed rat aorta evoked by different contractors including phenylephrine (Phe), KCl, and U46619 (9,11-dideoxy-9α,11α-methanoepoxy Prosta-glandin F2α). Also, the underlying mechanisms were involved in PI3K/Akt/eNOS/NO/cGMP signaling pathway, Ca^2+^ channels and K^+^ channels.

Hypertension is a common cardiovascular disease and the pathogenesis related to the abnormal vascular contractility and vasodilation. Normal endothelium function plays a vital role in the garden of vascular physiological homeostasis and vascular relaxation (Touyz, 2000; Zhou et al., 2017; Maruhashi et al., 2019). In the present study, we found that THP relaxed KCl, Phe, and U46619 induced vascular contraction and the relaxation effect was most effective in Phe-contracted rat aorta (Figure 1B and Table 1). Also, the pre-treatment of β-adrenergic receptor blocker propranolol, angiotensin II receptor blocker losartan and PD123319 or angiotensin converting enzyme inhibitor enalapril enhanced THP-induced vascular relaxation (Figure 1C and Table 1). THP did not affect the vascular contraction induced by angiotensin II (Figure 1D). These results indicated that THP significantly relaxed vascular aorta and the underlying mechanism was not involved in β-adrenergic receptor blockade and RAS inhibition.

Endothelium dependent relaxing factors (EDRF) including nitric oxide (NO) and prostacyclin (PGl_2_) play key roles in the maintenance of the normal physical tension and relaxation function of vessel (Vanhoutte, 1989). NO was synthesized by endothelial nitric oxide synthase (eNOS) while cyclooxygenase-2 (COX2) and prostacyclin synthase (PGIS) mediated the production of PGI_2_ in endothelial cells (Dorris and Peebles, 2012). We found the vasorelaxation effect of THP was partially inhibited by eNOS inhibitor L-NAME in intact rat aorta regardless of COX2 inhibitor indomethacin (Indo). The inhibition effect of L-NAME + Indo was consistent to mechanical removal of endothelium (-endo) (Figure 2A and Table 1). Cyclic guanosine monophosphate (cGMP) which is generated by guanylyl cyclase mediates the bioavailability of NO and drives NO-dependent vascular relaxation in smooth muscle cells (Furchgott and Vanhoutte, 1989). Pre-treatment of guanylyl cyclase inhibitor ODQ and methyl blue (MB) also reduced the vascular relaxation effect of THP. In addition, L-arginine (L-Arg) which has higher binding ability to eNOS than L-NAME reversed the inhibition effect of L-NAME in THP-induced vascular relaxation. Combination of L-NAME and ODQ significantly inhibited THP-induced vascular relaxation (Figure 2C). PI3K/Akt activates the phosphorylation of eNOS and subsequently generates NO in endothelium. Their inhibitors wortmannin (Wort) and Akt inhibitor IV (Akti IV) slightly suppressed the vascular relaxation effect of THP (Figure 2D and Table 1). In addition, THP induced intracellular NO accumulation in HUVECs (Figure 2E,F). Thus, we could conclude that the underlying mechanism of THP-induced vascular relaxation was endothelium-dependently and involved in the PI3K/Akt/eNOS/NO/cGMP signaling pathway rather than COX2/PIG_2_. A previous study also reported that L-THP increased protein expression of phosphatidylinositol 3-kinase (PI3K) and phosphorylation of Akt and eNOS in myocardium (Han et al., 2012). So, PI3K/Akt/eNOS/NO/cGMP signaling pathway seems like an important pharmacological target of THP in cardiovascular disease.

The results in Figure 2A and Table 1 shown denuding aorta endothelium reduced the Emax of THP from 98.97 ± 1.31 to 85.58 ± 3.05% and indicated THP relaxed rat aorta mainly in an endothelium-independent manner. And the regulation of intracellular Ca^2+^ concentration by ion channels especially Ca^2+^ channels dramatically gardens vascular contraction and relaxation. We found THP inhibited the Phe or KCl induced vascular contraction concentration dependently (Figure 3A). And THP reduced CaCl_2_ triggered vascular tension of aorta in Ca^2+^-free depolarizing Krebs solution containing KCl or Phe (Figure 3B). Phe and KCl increased intracellular Ca^2+^ concentration by inducing extracellular Ca^2+^ influx and internal Ca^2+^ stores release (Niazmand et al., 2014; Zhou et al., 2017). In order to explore whether THP suppressed intracellular Ca^2+^ concentration, we measured the effect of THP on the cytosol Ca^2+^ amount change triggered by CaCl_2_ in Ca^2+^-free Krebs solution containing KCl in smooth muscle cells A7r5 by calcium imaging system. We found THP reduced the cytosol Ca^2+^ concentration in A7r5 cells (Figure 3C,D). L-type channel blocker nifedipine serve as positive control in these experiments. So, we could conclude that THP inhibited the vascular tension by blocking the Ca^2+^ influx. In addition, THP inhibited Phe and KCl-induced rat aorta contraction in the absence of extracellular Ca^2+^ concentration-dependently (Figure 4A,B). It seems that THP blocked the Ca^2+^ released from intracellular Ca^2+^ store and subsequently reduced the Phe and KCl induced transient vascular tension in Ca^2+^ free Krebs solution. Consistently, we found the inhibition of the IP3 and Ryr receptor which mediated the Ca^2+^ release from intracellular Ca^2+^ store attenuated the vascular relaxation effect of THP (Figure 4C and Table 2). Thus, we could conclude that THP reduced the intracellular Ca^2+^ release induced vascular tension by blocking IP3 and Ryr receptors. The Ca^2+^ and Ca^2+^/Calmodulin (CaM) complex activates smooth muscle myosin light chain kinase (MLCK) and induced calcium sensitization during vascular contraction (Martinsen et al., 2014; Zhou et al., 2017). However, THP relaxed PMA (Phorbol 12-myristate 13-acetate) which trigger calcium sensitization by active PKC and ROCK signaling pathway induced vascular contraction at the concentration of 300 μM (Supplementary Figure S1). THP also enhanced vascular relaxation effect of bradykinin and protein expression of bradykinin in endothelial cells (Supplementary Figure S2). So, THP relaxed vascular tension may also relate to the inhibition of intracellular calcium sensitization and the production of bradykinin.

Vascular tension and relaxation were also regulated by K^+^ channels. In this study, we found the voltage-dependent K^+^ channel (Kv) blocker 4-aminopyridine (4-AP), ATP-sensitive K^+^ channel (KATP) blocker glibenclamide (Gly), and inward rectifying K^+^ channel blocker (BaCl_2_) attenuated THP induced vascular relaxation in intact rat aorta rather than endothelium denuded rat aorta (Figure 5). Li et al. (2017) also found that THP inhibited the kinetic activity of Kv1.5 channels. Thus, we could conclude that THP relaxed rat aorta partially though K^+^ channels and endothelium also plays a key role.

However, this study has some limitations. Mas-receptor (MS-R) which presents opposite function to angiotensin II receptor type 1 plays a vital role in vasodilation (Katsi et al., 2018) and whether THP could activate MS-R is unknown. THP induced the production of bradykinin while the underlying mechanism needs to be further studied. Moreover, whether THP could lower the blood pressure *in vivo* and protect the hypertension-related endothelium dysfunction and other complications also needs to be studied in the future.

In summary, THP relaxed vessel contracted by different contractors including Phe, KCl, and U46619. The underlying mechanism was both endothelium-dependent and independent and was involved in the PI3K/Akt/eNOS/NO/cGMP signaling pathway as well as the Ca^2+^ and K^+^ channels. Thus, THP may be a potential treatment for cardiovascular disease like hypertension in clinics.

## Author Contributions

Z-YZ conducted the experiments related to vascular relaxation effect of THP and wrote the manuscript. W-RZ conducted the experiments about the mechanism of vascular relaxation effect of THP. W-TS conducted the cell culture and related experiments. YX, Z-LM, and L-QZ analyzed the data of these experiments. J-YT and J-GX designed the experiments. X-LC and QY designed the experiments and revised the manuscript.

## Conflict of Interest Statement

The authors declare that the research was conducted in the absence of any commercial or financial relationships that could be construed as a potential conflict of interest.
