# Supplementary material for: Endothelial-Dependent and Independent Vascular Relaxation Effect of Tetrahydropalmatine on Rat Aorta
Source: Front Pharmacol. 2019 Apr 16;10:336. doi: 10.3389/fphar.2019.00336 (PMC6477965; doi:10.3389/fphar.2019.00336)
Supplement: Supplementary file 1 [file Data_Sheet_1.docx]

**Supplemental data**


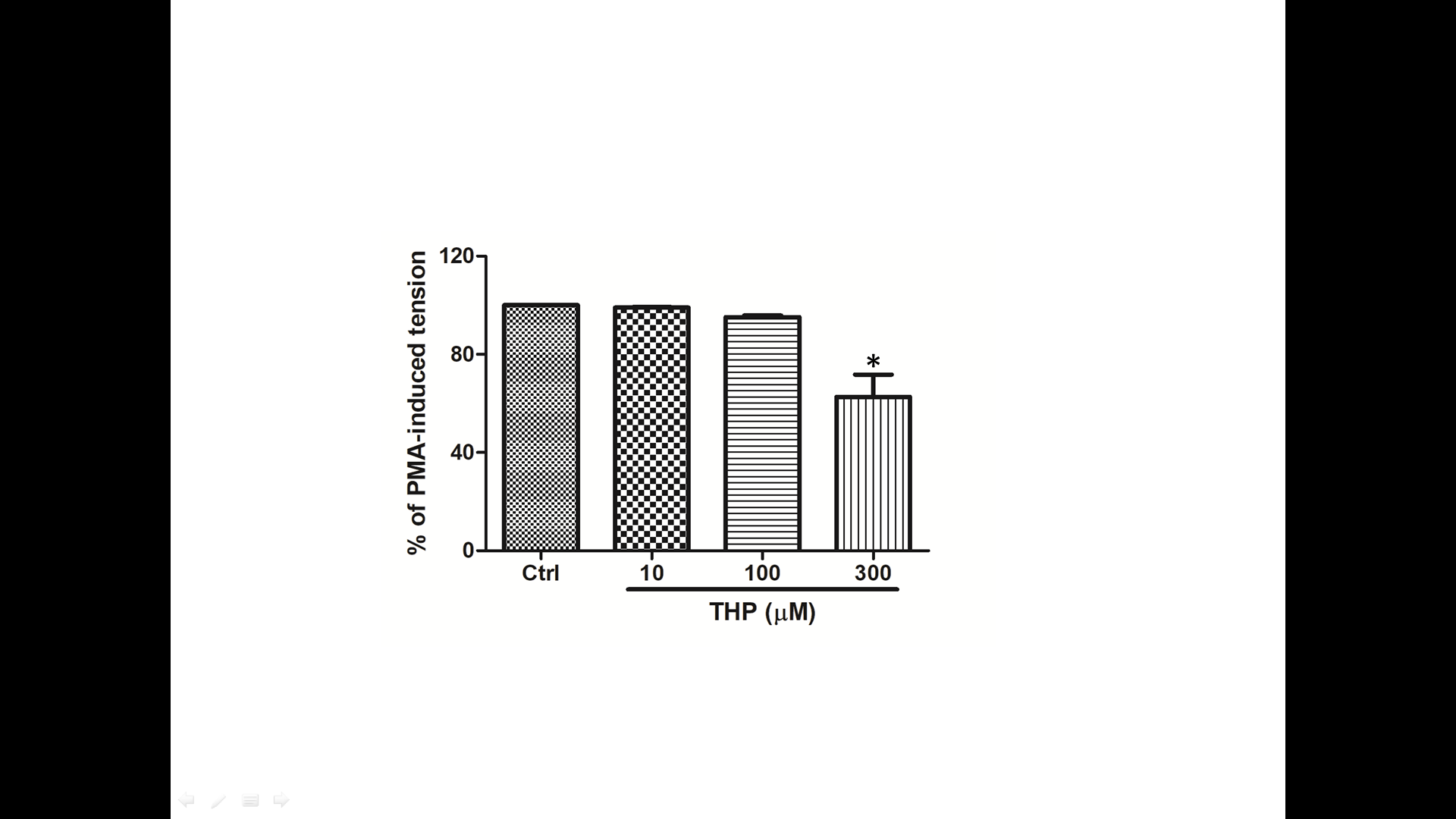


**Fig S1 THP relaxed PMA induced vascular relaxation in rat aorta**. In endothelial denuded rat aorta, PMA (Phorbol 12-myristate 13-acetate, 1µM) induced vascular contraction and THP only relaxed vascular at high concentration (300 µM).


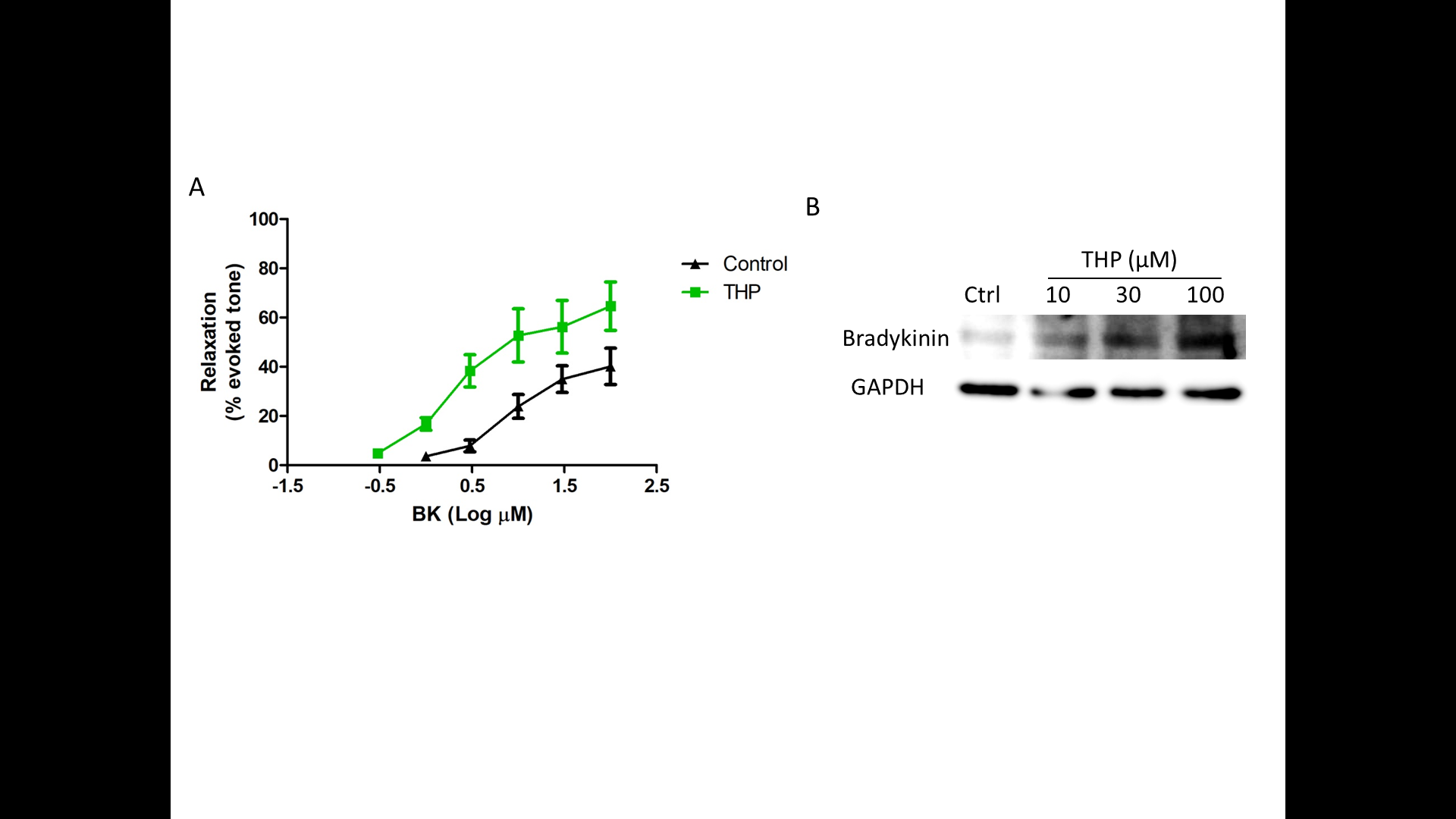


**Fig S2 The effect of THP on bradykinin induced vascular relaxation in rat aorta**. (A) Aorta rings were pre-treated THP (10µM) for 30min before Phe (1µM) induced contraction, then followed by the accumulative addition of bradykinin (BK). (B) HUVECs were treated with THP (10, 30, 100µM) for 30min then the protein expression of bradykinin was analyzed by western blotting.
